# Targeting Angiogenesis in Childhood Sarcomas

**DOI:** 10.1155/2011/601514

**Published:** 2010-12-09

**Authors:** Hemant K. Bid, Peter J. Houghton

**Affiliations:** Center for Childhood Cancer, Nationwide Children's Hospital, 700 Children's Drive, Columbus, OH 43205, USA

## Abstract

Angiogenesis and vasculogenesis constitute two processes in the formation of new blood vessels and are essential for progression of solid tumors. Consequently, targeting angiogenesis, and to a lesser extent vasculogenesis, has become a major focus in cancer drug development. Angiogenesis inhibitors are now being tested in pediatric populations whereas inhibitors of vasculogenesis are in an earlier stage of development. Despite the initial enthusiasm for targeting angiogenesis for treatment of cancer, clinical trials have shown only incremental increases in survival, and agents have been largely cytostatic rather than inducing tumor regressions. Consequently, the role of such therapeutic approaches in the context of curative intent for childhood sarcomas is less clear. Here we review the literature on blood vessel formation in sarcomas with a focus on pediatric sarcomas and developments in targeting angiogenesis for treatment of these rare cancers.

## 1. Introduction

The generation of new capillaries from preexisting blood vessels is termed angiogenesis [1]. Angiogenesis functions as the result of a dynamic balance between proangiogenic factors, for example, vascular endothelial growth factor (VEGF) and platelet-derived growth factor (PDGF), and factors that inhibit angiogenesis such as thrombospondin-1 and angiostatin [2]. The process of regulated angiogenesis occurs during embryogenesis, the menstrual cycle, wound healing, and pathologic states. Unregulated angiogenesis may lead to numerous diseases and is thought to play an indispensable role in solid tumor growth and metastasis. Numerous investigations on tumor development have shown that an alteration in the blood supply can noticeably influence the tumor growth and its metastasis [2]. As with normal tissue, the growing tumor requires an extensive network of capillaries to provide the necessary nutrients and oxygen. Moreover, the new intratumoral blood vessels offer a way for tumor cells to enter the circulation and metastasize to distant organs. In this context, angiogenesis plays a crucial role in facilitating the growth of the primary tumor and generating metastasis. However, in the early 1900s it was recognized that “vessels showed changes, such as defective coatings, dilation, obliteration, and thrombosis” [3, 4] (cited in [5]). Extensive research in this area has indicated that the effective inhibition of blood vessel formation can result in tumor regression, although the predominant effect is the slowing of tumor growth. However, targeting the stromal elements of the tumor, rather than focusing on the cancer cells exclusively, represents a major shift in emphasis in cancer research. Unfortunately, due to the heterogeneity of the angiogenesic process within diverse neoplasms, it is difficult to generalize research findings to all tumor types. Here we have focused on the available data on angiogenesis and targeting angiogenesis as it pertains to pediatric sarcoma.

## 2. Angiogenesis in Childhood Sarcomas

Sarcomas present a great challenge for cancer therapy because they comprise a relatively uncommon group of diseases. Sarcomas encompass many diseases, not simply a representation of a single entity of mesenchymal origin. Pediatric soft tissue sarcomas are a group of malignant tumors that originate from primitive mesenchymal tissue and account for 7% of all childhood tumors [6]. As a result of their diverse biology, therapeutics for pediatric sarcomas will ultimately be tailored to the specific tissue type [7–10].

Established chemotherapy regimens for advanced or metastatic sarcoma generally have low 5-year event-free survival, and current therapies have substantial toxicity. Resistance often arises quickly, making advanced sarcoma an acceptable target for alternative treatment approaches. Antiangiogenic therapies have a number of potential advantages including decreased resistance, fewer side effects, and a broad spectrum of activity. Human sarcomas express a number of proangiogenic factors that may represent potential therapeutic targets, with VEGF being the best characterized. Inhibitors of angiogenesis have demonstrated antitumor activity in animal models of childhood sarcomas, and clinical trials are in the early stages, although promising results are already being seen. Antiangiogenic and immunomodulatory therapies are gaining momentum in the pediatric arena and, when tested in combination with traditional cytotoxic agents for recurrent and high-risk primary pediatric sarcomas, may lead to more effective and tolerable therapies [11].

An example of potential antiangiogenic therapeutic targets can be observed in rhabdomyosarcoma (RMS) cell lines. These cells secrete VEGF [12, 13] as well as other angiogenic factors such as basic fibroblastic growth factor (bFGF) and interleukin 8 [14] as well as other potential angiogenic factors [15]. In most RMS cell lines VEGF stimulates proliferation or activates the PI3K/Akt pathway [12, 13], hence acting as both an autocrine growth factor and a paracrine factor involved in angiogenesis.

Microvessel density (MVD) has also been found to be a prognostic factor in the response to therapy and survival in several adult carcinomas [16–19]. Observations from different studies suggest that MVD in soft tissue sarcomas (STS) was not associated with histological type, grading, metastatic behavior, or survival [20–23]. Rather, tissue levels of VEGF were associated with local recurrence and survival [20]. In contrast MVD was correlated with survival in adult soft tissue sarcoma of the extremities [24]. Tomlinson et al. describe a different pattern of angiogenesis in STS compared to breast carcinoma. In breast cancer, the capillaries were clustered in bursts within the stroma of the tumor, while the sarcoma capillaries were homogeneously distributed throughout the tumor stroma. They credit this difference to the greater number of activated fibroblasts in carcinomas, with their own gradients of angiogenic factors in the tissues. This aspect has been studied in carcinosarcoma, which contains both tumor types. In accordance with the findings of Tomlinson et al. [22], a study by Yoshida et al. describes a significantly higher MVD in the carcinomatous areas of the tumor than that found in the sarcomatous parts [25]. However, the consequences of this in terms of antiangiogenesis therapy of STS are not yet clear.

## 3. Angiogenic Factors Secreted by Childhood Sarcomas

As noted above, balance between proangiogenic and antiangiogenic factors, with the involvement of different cells and stimulating factors, regulates the process of angiogenesis. Some of the cells engaged are endothelial cells (EC), lymphocytes, macrophages, and mast cells. Vascular endothelial growth factor and fibroblast growth factor (FGF) are two of the major factors involved in this process. These cells and stimulating factors play different and important roles during tumor angiogenesis. From the more than 20 proangiogenic and antiangiogenic agents identified, VEGF and bFGF have been established as the two most potent positive regulators of angiogenesis [26–28]. However, other cytokines, such as interleukin-1 receptor *α* (IL-1R), IL-6, and IL-8, tumor growth factor-*α* (TGF-*α*), TGF-*β*, tumor necrosis factor-*α* (TNF-*α*), hepatocyte growth factor (HGF), leptin, and platelet-derived growth factor (PDGF), have been reported to be involved as well, though their roles have not always been clearly defined [28–34]. 

As previously described, tumor cells require nutrients and oxygen to overcome hypoxia and starvation. When a condition such as hypoxia is present within the tumor tissue, the tumor cells are stimulated to promote the secretion of various angiogenic factors for the induction of angiogenesis [35]. Of clinical significance, the pretherapeutic serum VEGF levels were found to be significantly higher in patients with osteosarcoma who relapsed during the first year of treatment, providing the basis to establish further antiangiogenic therapy to target patients at high risk of angiogenesis-dependent relapse of osteosarcoma [36]. However, the prognostic significance of angiogenic factors in childhood sarcoma remains ambiguous.

## 4. Antibody-Based Antiangiogenic Therapy

Bevacizumab (Avastin; Genentech Inc.), a humanized antibody against that binds VEGF [37], must be regarded as the gold standard against which other antiangiogenic treatments are compared. Bevacizumab specifically binds to VEGF-A and its isoforms to counteract the proangiogenic effects of VEGF, allowing for the normalization of the tumor vasculature [38]. It is approved for several adult indications and currently being evaluated in combination treatment regimens for various adult malignancies [39–41]. The clinical experience with bevacizumab in pediatric patients is limited. A report by Benesch et al. [42] indicated that bevacizumab has activity in pediatric malignancies, but large multicenter trials are needed to quickly assess the clinical value of this drug in childhood malignancies. Ongoing trials include evaluation against osteosarcoma and Malignant Fibrous Histiocytoma (MFH) of bone. Although bevacizumab is not without toxicity, pediatric trials combining this agent with conventional chemotherapy regimens are in development.

## 5. Small Molecule Inhibitors of Angiogenesis in Sarcomas

There are multiple humoral factors involved in the regulation of both normal and abnormal angiogenesis. From the perspective of small molecule development VEGF signaling has been the predominant target, as VEGF was found to be overexpressed in various malignancies [43, 44]. Preclinical studies have evaluated a wide range of strategies and compounds to inhibit angiogenesis in laboratory models. Of these agents, tyrosine kinase inhibitors (TKIs) have gained attention as a means of targeted treatment for a wide range of human cancers. At least 90 tyrosine kinase genes have been identified in human cancers [45], and several TKIs inhibitors are now approved for use in the treatment of cancer in the United States. However, only few of these have been adequately evaluated in childhood sarcoma. However “selective,” small molecule inhibitors usually do not inhibit only a single kinase, but result in the targeting multiple signaling pathways. The “multitargeted” TKIs have, in general, shown the most activity against solid tumors.

Of the small molecule inhibitors with oral bioavailability, sorafenib (Nexavar; Bayer Pharmaceuticals) and sunitinib (Sutent; Pfizer Inc.) act on multiple intracellular and receptor protein kinases (e.g., VEGF receptors, PDGFR, FLT3, RET, BRAF, KIT) that are components of signaling pathways controlling tumor growth and angiogenesis. Both of these agents have similar drug profiles and overlapping targets [46] and are currently approved by the U.S. Food and Drug Administration for the treatment of advanced renal cell carcinoma (RCC) in adults [47]. In a study conducted by the Pediatric Preclinical Testing Program (PPTP), sorafenib was demonstrated to be an effective inhibitor of tumor growth across multiple histotypes in vivo [48]. Currently, sorafenib is being evaluated in high-grade osteosarcoma. Sunitinib also showed significant tumor growth inhibition against most of the PPTP's solid tumor panels, but little activity against the neuroblastoma and ALL panels. The antitumor activity of sunitinib was manifested as primarily a delay in tumor growth, consistent with an antiangiogenic effect against many of the pediatric preclinical models evaluated [49].

Cediranib (Recentin; AZD2171; Astrazeneca Inc.), another small molecule with oral bioavailability, is an indole-ether quinazoline which inhibits the tyrosine kinase activity of the vascular endothelial growth factor receptors VEGFR-1 (Flt-1), VEGFR-2 (KDR), VEGFR-3 (Flt-4), and c-KIT. Cediranib was previously shown to prevent both physiologic and pathologic angiogenesis in vivo [50], inhibiting the growth of a number of different pediatric human tumor xenografts [50, 51]. Currently in phase II/III clinical development, early clinical studies have shown encouraging antitumor activity in patients with a broad range of solid tumors, as well as time-dependent and dose-dependent changes in pharmacodynamic markers [52]. The results from a recently completed clinical trial have shown that the daily administration of cediranib to glioblastoma patients resulted in a rapid and prolonged normalization of the tumor vasculature which subsequently led to a reduction in tumor-associated edema [53]. Promising preliminary results have also been reported for treatment of alveolar soft part sarcoma (ASPS), a tumor that responds poorly to conventional chemotherapeutic regimens [54]. Of seven patients with ASPS four had partial responses and two either marginal response or stable disease [55].

Rapamycin and its derivatives (temsirolimus, everolimus, radiforolimus) selectively inhibit a serine/threonine kinase mTOR (mammalian target of rapamycin) that controls translation and transcription. These are immunosuppressive macrocyclic lactone antibiotics that block mTOR function and produce an antiproliferative effect in a variety of malignancies. Rapamycin has demonstrated broad antitumor activity against the pediatric cancers in PPTP's in vivo tumor panels, with noteworthy activity in selected sarcoma and ALL xenografts [56]. Initial reports have suggested that the effects of rapamycin may be related to its inhibitory action against the endothelial cells, effectively blocking tumor angiogenesis [57, 58]. Rapamycin has the potential to disrupt the action of vascular endothelial growth factor (VEGF) in the growth plate and interfere with insulin-like growth factor I (IGF-I) signaling [59]. Rapalogs have been shown to inhibit hypoxia-induced induction of hypoxia inducible factor-1*α* (HIF-1*α*), the major transcription factor that reprograms cells under hypoxic stress and induces VEGF transcription. These new findings suggest potential benefits of including rapamycin as an antiangiogenic agent in the treatment regimens of pediatric cancer patients. However, at least in some sarcoma xenograft models, rapamycin treatment stimulated tumor-associated VEGF, although the mechanism for this is poorly understood [60].

## 6. Role of IGFs in Childhood Sarcomas

It is becoming increasingly evident that the Type-1 insulin-like growth factor (IGF-1R) and its ligands (IGF-1, IGF-2) play roles in both tumor cell proliferation and survival, and proliferation of vascular endothelial cells. IGFs are balanced by insulin-like growth factor-binding proteins (IGFBPs). IGFBPs comprise a family of secreted proteins that modulate the bioavailability of insulin-like growth factors (IGFs) in the IGF-I/IGF-I receptor (IGF-IR) signaling axis. IGF binding protein-3 (IGFBP-3) is emerging as a key regulator of cell growth and apoptosis, both as an IGF antagonist and as an independent molecule, which plays roles in the proliferation and migration of HUVEC cells. IGFBP-3 expression is generally inhibited in Ewing's sarcoma cells, as a consequence of EWS/FLI1 expression. Exposure of neoplastic cells to IGFBP-3 inhibits their growth, migratory, invasive, angiogenic, and metastatic potential, therefore demonstrating the protein as a molecule of therapeutic relevance to be considered in the treatment of patients with Ewing sarcoma [61]. In contrast, the IGFBP-3-induced endothelial cell motility and migration may suggest a direct role for this binding protein in promoting angiogenesis [62, 63]. The functional role of IGFBP-5 in retarding angiogenesis has also been described. Tumor growth and tumor vascularity were decreased in the presence of IGFBP-5 expression in a xenograft model of human ovarian cancer [64]. Insulin-like growth factor-binding protein-7 (IGFBP7) is a secreted 31-kDa protein, which is also called as IGFBP-related protein 1 (IGFBP-rP1) [65]. IGBP7 shares high homology with the IGFBPs and binds IGF-I and insulin, but its binding affinity for IGF-I is lower than those of IGFBPs 1 to 6 [66]. IGFBP7 is highly expressed in the blood vessels of various human cancer tissues, suggesting that it might suppress the pathological action of VEGF, which is mainly derived from tumor cells. Data suggest that IGFBP7 in the blood vessels of tumors may lead to a unique tumor vasculature with characteristics significantly different from those of normal vasculature. The inhibitory effect of IGFBP7 on tumorigenicity might be partially mediated by its ability to suppress VEGF-stimulated angiogenesis, although there is so far no direct evidence to explain if IGFBP7 affects tumor blood vessels. The use of IGFBPs to limit IGF-1R signaling has been proposed as a therapeutic approach. Gallicchio et al. [67] reported that IGFBP-6 significantly inhibited monolayer RD and Rh-30 cell proliferation in a dose-dependent manner. Furthermore, the overexpression of IGFBP-6 resulted in a 74–88% reduction in Rh-30 tumor size in vivo after 18 days, showing that IGFBP-6 can be a potent antitumor agent. 

For approximately two decades, the insulin-like growth factor (IGF) has been implicated in the pathogenesis of numerous pediatric malignancies, including osteosarcoma, Ewing sarcoma, and rhabdomyosarcoma (RMS). The role of IGF-1R signaling in the pathogenesis of RMS and its role in preventing apoptosis induced by a multitude of cellular stresses, including cytotoxic drugs, radiation, and hypoxia [68], indicate that targeting this pathway may have considerable utility in the therapy of RMS. IGF-II is also involved in normal muscle growth, and Northern blot analysis of tumor biopsy specimens from patients with both alveolar and embryonal rhabdomyosarcoma has demonstrated high levels of IGF-II mRNA expression [69]. This suggests the possibility that upregulation of IGF-II plays a role in the unregulated growth of these tumors. Support for this hypothesis came from the finding that RMS cell lines also secrete IGF-II, which then binds to IGF-1R, resulting in autocrine growth proliferation and increased cell motility [70].

Ewing's sarcoma, peripheral primitive neuroectodermal tumor, and Askin tumor form a group of tumors collectively termed Ewing's sarcoma family of tumors (ESFT). These tumors are characterized by specific chromosomal translocations that cause the N-terminus of EWS to be fused to the C-terminus of one member of the ETS family of transcription factors, most commonly FLI1 [71]. Expression of the fusion product has been implicated in oncogenesis. The role of IGF-1R signaling in ESFT has undergone extensive evaluation. EFST cell lines express IGF-1R and secrete IGF-I, and IGF-1R-blocking antibodies interrupt this autocrine loop [72, 73].

The importance of the IGF axis to cell growth and differentiation in both normal tissues and cancer and the aforementioned association of osteosarcoma with periods of rapid bone growth help to explain the current focus for therapies targeting the type 1 IGF receptor (IGF-1R). The peak incidence of osteosarcoma occurs during adolescence, corresponding to both the growth spurt and peak concentrations of circulating GH and IGF-1 [74]. This epidemiological correlation has led to the hypothesis that high levels of IGF-1 play an important role in the pathogenesis of osteosarcoma. This hypothesis is supported by a host of preclinical data: (a) osteosarcoma cells express functional IGF-1R on the cell surface, (b) exogenous IGF-1 stimulates osteosarcoma cells to proliferate, (c) IGF-1-dependent growth can be inhibited using monoclonal antibodies or antisense oligonucleotides against IGF-1R [75], (d) the treatment of mice with a humanized anti-IGF-1R antibody resulted in tumor regression in two osteosarcoma xenograft models [76], and (e) the majority of osteosarcoma patient samples express IGF ligands, and 45% express IGF-1R [77].

## 7. Role of IGF's in Angiogenesis

As described above, IGFBPs have both antitumor and antiangiogenic properties, although whether these two characteristics are linked remains to be demonstrated. However, these data suggest that antibodies that prevent ligand binding to the IGF-1R, or ligand binding antibodies *per se*, may have therapeutic utility in childhood sarcomas. Phase-1 or -2 clinical trials with eight fully human antibodies, or humanized antibodies, that target IGF-1R and prevent ligand binding have been reported [78]. For commercial reasons two agents (SCH717454 and R1507) are not being developed further. These antibodies show specificity for the IGF-IR although they may also inhibit chimeric receptors formed through heterodimerization with the insulin receptor. In preclinical models of childhood cancers, the prototypical anti-IGF-1R antibody, *α*-IR3, mediated downregulation of IGF-IR, significantly retarded the growth of several cell lines in vitro [70], and inhibited the growth of pediatric cancer xenografts [79]. SCH717454 significantly inhibits growth of RMS xenografts and induces regressions in several sarcoma histotypes, notably osteosarcoma and Ewing sarcoma [80]. R1507 was found to inhibit growth of osteosarcoma xenografts [81]. In some in vivo models of Ewing sarcoma and osteosarcoma targeting IGF-1R with CP751871 dramatically suppressed VEGF transcription and reduced tumor-associated VEGF within 24 hours of antibody administration [60]. Furthermore, SCH717454 treatment markedly reduced blood vessel formation in tumor xenografts, showing that the in vivo activity is derived not only from its inhibition of tumor cell proliferation, but also from its angiogenesis activity [82].

The molecular characterization of these sensitive models where IGF-IR signaling appears to be critical could identify subsets of tumors that have become “addicted” to this pathway [83]. In other preclinical models, blocking IGF-IR signaling results in significant retardation of tumor growth, although in a clinical setting this response would still be scored as progressive disease. In these models with intermediate sensitivity, such as RMS, combinations of signaling inhibitors would be a potentially more effective antitumor therapy. One strategy that is being evaluated in preclinical models is the combination of the mTOR inhibitor, rapamycin, with IGF-1R inhibitors. The basis for this combination is that inhibition of mTOR upregulates IGF-1R signaling through stabilization of IRS-1 [84] and IGF-1R signaling blocks rapamycin-induced apoptosis [85, 86]. One IGF-1R inhibitor, CP751871, caused complete IGF-1R downregulation, suppressed AKT phosphorylation, and dramatically suppressed tumor-derived vascular endothelial growth factor (VEGF) in some sarcoma xenografts. Treatment with rapamycin alone did not markedly suppress VEGF in tumors and synergized only in those tumor lines where VEGF was inhibited by CP751871. This data suggests a model in which the blockade of IGF-1R suppresses tumor-derived VEGF to a level where rapamycin can effectively suppress the response in vascular endothelial cells [60]. Exactly how rapamycin blocks the response to VEGF in vascular endothelial cells is not clear. However, recent studies show that SCH717454 potently inhibits VEGF-induced proliferation of HUVECs, indicating that IGF-1R-mediated signaling is essential for vascular endothelial cell proliferation (H. K. Bid, PJH, unpublished data). As discussed above, rapamycin has been shown to potently inhibit IGF-1-stimulated proliferation of tumor cells [87]. 

Alternative approaches to inhibiting IGF-1R signaling comprise the development of ligand binding antibodies. Targeting the receptor ligand rather than the receptor *per se* has proven to be a valuable approach for the antiangiogenic antibody (bevacizumab), and high-affinity, fully human antibodies have been developed against IGF-II [88].

Phase I and phase II trials using many of these IGF-1R-targeted antibodies are currently in progress. To date, there have been very few serious side effects resulting from this treatment. Hyperglycemia, when present, has been mild and has only been seen in some of the antibodies tested [89–93]. Because of the important role of the IGF pathway in normal growth, there is concern about the impact of IGF blockade in patients who are still growing. Details of recent clinical trials are provided in Table 1. Unfortunately, in these tumor types, many of the patients are teenagers or younger children. The hypothetical concern of disrupting normal growth must be taken under consideration, but also weighed against the pressing issue of tumor progression. The only way to assess the impact of IGF-1R-directed therapy on normal growth is to monitor young patients who have been treated with the antibody for a prolonged period over the course of their growth. On the positive side, this would indicate that the patient is responding or the disease is not progressing, on therapy. Data have not emerged suggesting that one antibody is more effective than another. However, slight differences in regards to whether the antibody is fully human or humanized, their relative affinity to the IGF-1R and IR, and their ability to block the binding of either IGF-I or IGF-II ligand have not resulted in markedly different side effects or tolerability, but could lead to differences in clinical activity.

## 8. Vasculogenesis in Childhood Sarcoma

Vasculogenesis and angiogenesis are the fundamental processes by which new blood vessels are formed [35, 94, 95]. Vasculogenesis is defined as the differentiation of precursor cells (angioblasts) into endothelial cells and the de novo formation of a primitive vascular network, whereas angiogenesis is defined as the growth of new capillaries from preexisting blood vessels [35]. Several studies have now been published that suggest that not only angiogenesis but also vasculogenesis may be involved during postnatal life in situations that require an expanded vessel network. Solid tumors require development and expansion of a vascular network for nourishment to support their growth. Angiogenesis was initially regarded as the sole mechanism by which tumor vessels expand. However, other mechanisms are also involved in the expansion of the tumor vascular network such as vasculogenesis. 

Both angiogenesis and vasculogenesis contribute to the formation and expansion of the tumor vasculature that supports the growth of Ewing's sarcoma [96]. Data from Lee et al., 2006 [96] support the same hypothesis because they demonstrated that not only local endothelial cells but also bone marrow (BM)-derived cells are involved in the generation of the new tumor vasculature during the growth of Ewing's sarcoma. Several cytokines, such as Granulocyte Colony-Stimulating Factor, Placental Growth Factor, and Stromal cell-derived Factor-1 (SDF-1), have been shown to induce BM stem/progenitor cell mobilization and chemotaxis. With respect to tumor growth, both stimulatory and inhibitory roles of SDF-1 have been reported. Disruption of the SDF-1/CXCR4 axis was found to inhibit tumor growth, microvessel density, and intratumoral blood flow without affecting VEGF levels [97]. Reddy et al., 2008 [98] suggest that the effects of SDF-1 on tumor neovascularization include augmented chemotaxis of BM cells, retainment of BM-derived pericytes in close association with the vessel endothelial lining, enhanced overall pericyte coverage of tumor neovessels, and remodeling of vascular endothelium into larger, functional structures. All these processes together support the growth of Ewing's tumors, with distinctly reduced VEGF_165_. Overall these reports suggest that BM-derived cells play a critical role in the expansion of the Ewing's tumor vasculature, and that vasculogenesis may be one of the mechanism by which tumors can evade the effects of antiangiogenic therapy targeted at VEGF. Similarly, vasculogenesis is likely in other sarcomas. With inhibitors of CXCR4 in development, the therapeutic potential for simultaneous inhibition of angiogenesis and vasculogenesis may be tested.

## 9. Summary and Perspective

Growth of sarcoma, like other solid tumors, is dependent on angiogenesis and vasculogenesis, hence understanding the basic mechanisms and factors that influencing these processes has the potential to reveal additional targets for intervention. To date, the effectiveness of angiogenesis-directed treatments has not been particularly striking. In adults these agents extend event-free survival by weeks or months in the majority of malignancies. Thus, the role of such therapeutic approaches must be considered in the context of childhood cancer where the intent is cure. One can see the value of essentially cytostatic therapy in the context of young children where delay in tumor progression may be of value in delaying radiation treatment (particularly in CNS malignancies). However, is it realistic to anticipate that antiangiogenic treatments will convert childhood cancer into a chronic disease? Genomic plasticity is the hallmark of cancer, thus one would anticipate evolution of cancer cells that circumvent such treatments. Thus, the role of antiangiogenic therapy is most likely in the context of conventional chemotherapy, or in combination with other signaling inhibitors. Whether addition of small molecule inhibitors of angiogenesis will permit the maintenance of dose intensity of current cytotoxic regimens remains to be determined. For example, results from adult clinical trials suggest that dose intensity of cytotoxic agents or the dose of cediranib has to be reduced [99]. Available results from trials of IGF-1R-targeted antibodies suggest a low response rate even in Ewing sarcoma, suggesting that these agents poorly suppress tumor cell proliferation or tumor cells are able to circumvent the antiangiogenic activity of these antibodies. One aspect of antiangiogenic therapy that holds some promise is the effect of vascular normalization that allows reoxygenation [100, 101] (and hence increased radiation sensitivity) or increased uptake of drugs into tumor tissue [102–104].

## Figures and Tables

**Table 1:** Summary of select recent clinical studies for sarcoma.

Compounds/Agents in Clinical trials	Tumor type	Identifier
A Phase II trial of dasatinib	Advanced sarcoma	SARC009
A Phase II trial of R1507, a recombinant human monoclonal antibody to the insulin-like growth factor-1 receptor	Recurrent or refractory ewing's sarcoma, osteosarcoma, synovial sarcoma, rhabdomyosarcoma, and other sarcomas	SARC011
A randomized, double-blinded, placebo-controlled, multiinstitutional, phase II.5 study of AZD0530, a selective Src kinase inhibitor	Recurrent osteosarcoma localized to the lung	SARC012
IMC-A12 and doxorubicin hydrochloride	Patients with unresectable, locally advanced, or metastatic soft tissue sarcoma	NCT00720174
IMC-A12	Young patients with relapsed or refractory ewing sarcoma/peripheral primitive neuroectodermal tumor or other solid tumor	NCT00609141
R1507	Patients with recurrent or refractory sarcoma	NCT00642941
CP751871 figitumumab combined with pegvisomant	Advanced solid tumors	NCT00976508
Bevacizumab and AZD2171	Patients with metastatic or unresectable solid tumor, lymphoma, intracranial glioblastoma, gliosarcoma, or anaplastic astrocytoma	NCT00458731
*Cediranib* (tentative trade name recentin), also known as AZD2171	Young patients with refractory or recurrent solid tumors or acute myeloid leukemia	NCT00354848
Temozolomide, cixutumumab, and combination chemotherapy	Treating patients with metastatic rhabdomyosarcoma	NCT01055314
Cixutumumab IMC A12	Treating patients with relapsed or refractory solid tumors	NCT00831844
Cixutumumab and temsirolimus	Treating young patients with solid tumors that have recurred or not responded to treatment	NCT00880282
A study of SCH 717454 in combination with different treatment regimens	Pediatric subjects with advanced solid tumors (Study P05883)	NCT00960063
SCH 717454 in combination with different treatment regimens	Advanced solid tumors (P04722)	NCT00954512
Temsirolimus and valproic acid	Treating young patients with relapsed neuroblastoma, bone sarcoma, or soft tissue sarcoma	NCT01204450
Temsirolimus, irinotecan hydrochloride, and temozolomide	Treating young patients with relapsed or refractory solid tumors	NCT01141244
PCI-24781 in combination with doxorubicin	Treat sarcoma	NCT01027910
Angiogenesis inhibitor SU5416	Treating patients with soft tissue sarcoma	NCT00023738
Sorafenib and bevacizumab	Treating patients with refractory, metastatic, or unresectable solid tumors	NCT00098592
Sunitinib	Treating patients with metastatic, locally advanced, or locally recurrent sarcomas	NCT00474994
Radiation therapy with or without SU5416 (TK inhibitor antiangiogenesis compound)	Treating patients with soft tissue sarcoma	NCT00023725
Phase II study of doxorubicin and bevacizumab (anti-VEFG monoclonal antibody, NSC 704865)	Patients with advanced or metastatic soft-tissue sarcoma	NCT00052390
Phase I/II study of gemcitabine, docetaxel, and bevacizumab	Patients with soft tissue sarcoma	NCT00276055
Phase II study of neoadjuvant bevacizumab and radiation therapy	Resectable soft tissue sarcomas	NCT00356031

*Source: http://www.clinicaltrials.gov/.

## References

[1] Lutsenko SV, Kiselev SM, Severin SE (2003). Molecular mechanisms of tumor angiogenesis. *Biochemistry*.

[2] de Castro Junior G, Puglisi F, de Azambuja E, El Saghir NS, Awada A (2006). Angiogenesis and cancer: a cross-talk between basic science and clinical trials (the "do ut des" paradigm). *Critical Reviews in Oncology/Hematology*.

[3] Borst M

[4] Ribbert H (1904). *Das Karzinom des Menschen sein Bau. seine Wachstum, seine Entstehung*.

[5] Goldacre RJ, Sylven B (1962). On the access of blood-borne dyes to various tumour regions. *British Journal of Cancer*.

[6] Dillon PW, Whalen TV, Azizkhan RG (1995). Neonatal soft tissue sarcomas: the influence of pathology on treatment and survival. *Journal of Pediatric Surgery*.

[7] Spunt SL, Skapek SX, Coffin CM (2008). Pediatric nonrhabdomyosarcoma soft tissue sarcomas. *Oncologist*.

[8] Pisters PWT, O’Sullivan B, Maki RG (2007). Evidence-based recommendations for local therapy for soft tissue sarcomas. *Journal of Clinical Oncology*.

[9] Anderson PM, Pearson M (2006). Novel therapeutic approaches in pediatric and young adult sarcomas. *Current Oncology Reports*.

[10] Ordóñez JL, Martins AS, Osuna D, Madoz-Gúrpide J, de Alava E (2008). Targeting sarcomas: therapeutic targets and their rational. *Seminars in Diagnostic Pathology*.

[11] Heymach JV (2001). Angiogenesis and antiangiogenic approaches to sarcomas. *Current Opinion in Oncology*.

[12] Kurmasheva RT, Harwood FC, Houghton PJ (2007). Differential regulation of vascular endothelial growth factor by Akt and mammalian target of rapamycin inhibitors in cell lines derived from childhood solid tumors. *Molecular Cancer Therapeutics*.

[13] Gee MFW, Tsuchida R, Eichler-Jonsson C, Das B, Baruchel S, Malkin D (2005). Vascular endothelial growth factor acts in an autocrine manner in rhabdomyosarcoma cell lines and can be inhibited with all-trans-retinoic acid. *Oncogene*.

[14] Pavlakovic H, Havers W, Schweigerer L (2001). Multiple angiogenesis stimulators in a single malignancy: implications for anti-angiogenic tumour therapy. *Angiogenesis*.

[15] De Giovanni C, Melani C, Nanni P (1995). Redundancy of autocrine loops in human rhabdomyosarcoma cells: induction of differentiation by suramin. *British Journal of Cancer*.

[16] Yamazaki K, Abe S, Takekawa H (1994). Tumor angiogenesis in human lung adenocarcinoma. *Cancer*.

[17] Maeda K, Chung YS, Takatsuka S (1995). Tumor angiogenesis as a predictor of recurrence in gastric carcinoma. *Journal of Clinical Oncology*.

[18] Drawer MK (1996). Quantitative microvessel density: a staging and prognostic marker for human prostatic carcinoma. *Cancer*.

[19] Gasparini G, Bonoldi E, Viale G (1996). Prognostic and predictive value of tumour angiogenesis in ovarian carcinomas. *International Journal of Cancer*.

[20] Yudoh K, Kanamori M, Ohmori K, Yasuda T, Aoki M, Kimura T (2001). Concentration of vascular endothelial growth factor in the tumour tissue as a prognostic factor of soft tissue sarcomas. *British Journal of Cancer*.

[21] Kawauchi S, Fukuda T, Tsuneyoshi M (1999). Angiogenesis does not correlate with prognosis or expression of vascular endothelial growth factor in synovial sarcomas. *Oncology Reports*.

[22] Tomlinson J, Barsky SH, Nelson S (1999). Different patterns of angiogenesis in sarcomas and carcinomas. *Clinical Cancer Research*.

[23] Saenz NC, Heslin MJ, Adsay V (1998). Neovascularity and clinical outcome in high-grade extremity soft tissue sarcomas. *Annals of Surgical Oncology*.

[24] Comandone A, Boglione E, Berardengo A Microvessel density (MVD) as a marker of neoangiogenesis: prognostic significance in correlation with grading and stage in adult soft tissue sarcomas (STS) of the extremities. A prospective study.

[25] Yoshida Y, Kurokawa T, Fukuno N, Nishikawa Y, Kamitani N, Kotsuji F (2000). Markers of apoptosis and angiogenesis indicate that carcinomatous components play an important role in the malignant behavior of uterine carcinosarcoma. *Human Pathology*.

[26] Dvorak HF (2002). Vascular permeability factor/vascular endothelial growth factor: a critical cytokine in tumor angiogenesis and a potential target for diagnosis and therapy. *Journal of Clinical Oncology*.

[27] Montesano R, Vassalli JD, Baird A (1986). Basic fibroblast growth factor induces angiogenesis in vitro. *Proceedings of the National Academy of Sciences of the United States of America*.

[28] Basilico C, Moscatelli D (1992). The FGF family of growth factors and oncogenes. *Advances in Cancer Research*.

[29] Fuhrmann-Benzakein E, Ma MN, Rubbia-Brandt L (2000). Elevated levels of angiogenic cytokines in the plasma of cancer patients. *International Journal of Cancer*.

[30] Pantschenko AG, Pushkar I, Anderson KH (2003). The interleukin-1 family of cytokines and receptors in human breast cancer: implications for tumor progression. *International Journal of Oncology*.

[31] Hatzi E, Murphy C, Zoephel A (2002). N-myc oncogene overexpression down-regulates IL-6; evidence that IL-6 inhibits angiogenesis and suppresses neuroblastoma tumor growth. *Oncogene*.

[32] Lin Y, Huang R, Chen L (2004). Identification of interleukin-8 as estrogen receptor-regulated factor involved in breast cancer invasion and angiogenesis by protein arrays. *International Journal of Cancer*.

[33] Zhang YUW, Su Y, Volpert OV, Vande Woude GF (2003). Hepatocyte growth factor/scatter factor mediates angiogenesis through positive VEGF and negative thrombospondin 1 regulation. *Proceedings of the National Academy of Sciences of the United States of America*.

[34] Brizzi MF, Battaglia E, Montrucchio G (1999). Thrombopoietin stimulates endothelial cell motility and neoangiogenesis by a platelet-activating factor-dependent mechanism. *Circulation Research*.

[35] Risau W (1997). Mechanisms of angiogenesis. *Nature*.

[36] Kaya M, Wada T, Akatsuka T (2000). Vascular endothelial growth factor expression in untreated osteosarcoma is predictive of pulmonary metastasis and poor prognosis. *Clinical Cancer Research*.

[37] Ferrara N, Hillan KJ, Gerber HP, Novotny W (2004). Discovery and development of bevacizumab, an anti-VEGF antibody for treating cancer. *Nature Reviews Drug Discovery*.

[38] Jain RK (2005). Normalization of tumor vasculature: an emerging concept in antiangiogenic therapy. *Science*.

[39] Yang JC, Haworth L, Sherry RM (2003). A randomized trial of bevacizumab, an anti-vascular endothelial growth factor antibody, for metastatic renal cancer. *New England Journal of Medicine*.

[40] Miller KD, Chap LI, Holmes FA (2005). Randomized phase III trial of capecitabine compared with bevacizumab plus capecitabine in patients with previously treated metastatic breast cancer. *Journal of Clinical Oncology*.

[41] Crane CH, Ellis LM, Abbruzzese JL (2006). Phase I trial evaluating the safety of bevacizumab with concurrent radiotherapy and capecitabine in locally advanced pancreatic cancer. *Journal of Clinical Oncology*.

[42] Benesch M, Windelberg M, Sauseng W (2008). Compassionate use of bevacizumab (Avastin) in children and young adults with refractory or recurrent solid tumors. *Annals of Oncology*.

[43] Wong C, Wellman TL, Lounsbury KM (2003). VEGF and HIF-1*α* expression are increased in advanced stages of epithelial ovarian cancer. *Gynecologic Oncology*.

[44] Han H, Silverman JF, Santucci TS (2001). Vascular endothelial growth factor expression in stage I non-small cell lung cancer correlates with neoangiogenesis and a poor prognosis. *Annals of Surgical Oncology*.

[45] Manning G, Whyte DB, Martinez R, Hunter T, Sudarsanam S (2002). The protein kinase complement of the human genome. *Science*.

[46] Kim A, Balis FM, Widemann BC (2009). Sorafenib and sunitinib. *Oncologist*.

[47] Grandinetti CA, Goldspiel BR (2007). Sorafenib and sunitinib: novel targeted therapies for renal cell cancer. *Pharmacotherapy*.

[48] Keir ST, Maris JM, Lock R (2010). Initial testing (stage 1) of the multi-targeted kinase inhibitor sorafenib by the pediatric preclinical testing program. *Pediatric Blood & Cancer*.

[49] Maris JM, Courtright J, Houghton PJ (2008). Initial testing (stage 1) of sunitinib by the pediatric preclinical testing program. *Pediatric Blood and Cancer*.

[50] Wedge SR, Kendrew J, Hennequin LF (2005). AZD2171: a highly potent, orally bioavailable, vascular endothelial growth factor receptor-2 tyrosine kinase inhibitor for the treatment of cancer. *Cancer Research*.

[51] Maris JM, Courtright J, Houghton PJ (2008). Initial testing of the VEGFR inhibitor AZD2171 by the Pediatric Preclinical Testing Program. *Pediatric Blood and Cancer*.

[52] Drevs J, Siegert P, Medinger M (2007). Phase I clinical study of AZD2171, an oral vascular endothelial growth factor signaling inhibitor, in patients with advanced solid tumors. *Journal of Clinical Oncology*.

[53] Batchelor TT, Sorensen AG, di Tomaso E (2007). AZD2171, a pan-VEGF receptor tyrosine kinase inhibitor, normalizes tumor vasculature and alleviates edema in glioblastoma patients. *Cancer Cell*.

[54] Reichardt P, Lindner T, Pink D, Thuss-Patience PC, Kretzschmar A, Dörken B (2003). Chemotherapy in alveolar soft part sarcomas: what do we know?. *European Journal of Cancer*.

[55] Gardner K, Judson I, Leahy M (2009). Activity of cediranib, a highly potent and selective VEGF signaling inhibitor, in alveolar soft part sarcoma. *Journal of Clinical Oncology*.

[56] Houghton PJ, Morton CL, Kolb EA (2008). Initial testing (stage 1) of the mTOR inhibitor rapamycin by the pediatric preclinical testing program. *Pediatric Blood and Cancer*.

[57] Humar ROK, Kiefer FN, Berns H, Resink TJ, Battegay EJ (2002). Hypoxia enhances vascular cell proliferation and angiogenesis in vitro via rapamycin (mTOR)-dependent signaling. *FASEB Journal*.

[58] Guba M, Von Breitenbuch P, Steinbauer M (2002). Rapamycin inhibits primary and metastatic tumor growth by antiangiogenesis: involvement of vascular endothelial growth factor. *Nature Medicine*.

[59] Álvarez-García Ó, García-López E, Loredo V (2010). Rapamycin induces growth retardation by disrupting angiogenesis in the growth plate. *Kidney International*.

[60] Kurmasheva RT, Dudkin L, Billups C, Debelenko LV, Morton CL, Houghton PJ (2009). The insulin-like growth factor-1 receptor-targeting antibody, CP-751,871, suppresses tumor-derived VEGF and synergizes with rapamycin in models of childhood sarcoma. *Cancer Research*.

[61] Benini S, Zuntini M, Manara MC (2006). Insulin-like growth factor binding protein 3 as an anticancer molecule in Ewing’s sarcoma. *International Journal of Cancer*.

[62] Granata R, Trovato L, Garbarino G (2004). Dual effects of IGFBP-3 on endothelial cell apoptosis and survival: involvement of the sphingolipid signaling pathways. *FASEB Journal*.

[63] Liu B, Lee KW, Anzo M (2007). Insulin-like growth factor-binding protein-3 inhibition of prostate cancer growth involves suppression of angiogenesis. *Oncogene*.

[64] Rho SB, Dong SM, Kang S (2008). Insulin-like growth factor-binding protein-5 (IGFBP-5) acts as a tumor suppressor by inhibiting angiogenesis. *Carcinogenesis*.

[65] Hwa V, Oh Y, Rosenfeld RG (1999). Insulin-like growth factor binding proteins: a proposed superfamily. *Acta Paediatrica*.

[66] Collet C, Candy J (1998). How many insulin-like growth factor binding proteins?. *Molecular and Cellular Endocrinology*.

[67] Gallicchio MA, Kneen M, Hall C, Scott AM, Bach LA (2001). Overexpression of insulin-like growth factor binding protein-6 inhibits rhabdomyosarcoma growth in vivo. *International Journal of Cancer*.

[68] Kurmasheva RT, Houghton PJ (2006). IGF-I mediated survival pathways in normal and malignant cells. *Biochimica et Biophysica Acta*.

[69] Minniti CP, Tsokos M, Newton WA, Helman LJ (1994). Specific expression of insulin-like growth factor-II in rhabdomyosarcoma tumor cells. *American Journal of Clinical Pathology*.

[70] El-Badry OM, Minniti C, Kohn EC, Houghton PJ, Daughaday WH, Helman LJ (1990). Insulin-like growth factor II acts as an autocrine growth and motility factor in human rhabdomyosarcoma tumors. *Cell Growth & Differentiation*.

[71] Owen LA, Lessnick SL (2006). Identification of target genes in their native cellular context: an analysis of EWS/FLI in Ewing’s sarcoma. *Cell Cycle*.

[72] Scotlandi K, Benini S, Sarti M (1996). Insulin-like growth factor I receptor-mediated circuit in Ewing’s sarcoma/peripheral neuroectodermal tumor: a possible therapeutic target. *Cancer Research*.

[73] Yee D, Favoni RE, Lebovic GS (1990). Insulin-like growth factor I expression by tumors of neuroectodermal origin with the t(11;22) chromosomal translocation. A potential autocrine growth factor. *Journal of Clinical Investigation*.

[74] Calle EE, Kaaks R (2004). Overweight, obesity and cancer: epidemiological evidence and proposed mechanisms. *Nature Reviews Cancer*.

[75] Kappel CC, Velez-Yanguas MC, Hirschfeld S, Helman LJ (1994). Human osteosarcoma cell lines are dependent on insulin-like growth factor I for in vitro growth. *Cancer Research*.

[76] Anders Kolb E, Gorlick R, Houghton PJ (2008). Initial testing (stage 1) of a monoclonal antibody (SCH 717454) against the IGF-1 receptor by the pediatric preclinical testing program. *Pediatric Blood and Cancer*.

[77] Burrow S, Andrulis IL, Pollak M, Bell RS (1998). Expression of insulin-like growth factor receptor, IGF-1, and IGF-2 in primary and metastatic osteosarcoma. *Journal of Surgical Oncology*.

[78] Feng Y, Dimitrov DS (2008). Monoclonal antibodies against components of the IGF system for cancer treatment. *Current Opinion in Drug Discovery and Development*.

[79] Kalebic T, Tsokos M, Helman LJ (1994). In vivo treatment with antibody against IGF-1 receptor suppresses growth of human rhabdomyosarcoma and down-regulates p34(cdc2). *Cancer Research*.

[80] Anders Kolb E, Gorlick R, Houghton PJ (2008). Initial testing (stage 1) of a monoclonal antibody (SCH 717454) against the IGF-1 receptor by the pediatric preclinical testing program. *Pediatric Blood and Cancer*.

[81] Kolb EA, Kamara D, Zhang W (2010). R1507, a fully human monoclonal antibody targeting IGF-1R, is effective alone and in combination with rapamycin in inhibiting growth of osteosarcoma xenografts. *Pediatric Blood and Cancer*.

[82] Wang Y, Lipari P, Wang X (2010). A fully human insulin-like growth factor-I receptor antibody SCH 717454 (robatumumab) has antitumor activity as a single agent and in combination with cytotoxics in pediatric tumor xenografts. *Molecular Cancer Therapeutics*.

[83] Weinstein IB, Joe AK (2006). Mechanisms of Disease: oncogene addiction—a rationale for molecular targeting in cancer therapy. *Nature Clinical Practice Oncology*.

[84] Easton JB, Kurmasheva RT, Houghton PJ (2006). IRS-1: auditing the effectiveness of mTOR inhibitors. *Cancer Cell*.

[85] Huang S, Shu L, Dilling MB (2003). Sustained activation of the JNK cascade and rapamycin-induced apoptosis are suppressed by p53/p21. *Molecular Cell*.

[86] Thimmaiah KN, Easton J, Huang S (2003). Insulin-like growth factor i-mediated protection from rapamycin-induced apoptosis is independent of Ras-Erk1-Erk2 and phosphatidylinositol 3′-kinase-Akt signaling pathways. *Cancer Research*.

[87] Dilling MB, Dias P, Shapiro DN, Germain GS, Johnson RK, Houghton PJ (1994). Rapamycin selectively inhibits the growth of childhood rhabdomyosarcoma cells through inhibition of signaling via the type I insulin-like growth factor receptor. *Cancer Research*.

[88] Feng Y, Zhu Z, Xiao X, Choudry V, Barrett JC, Dimitrov DS (2006). Novel human monoclonal antibodies to insulin-like growth factor (IGF)-II that potently inhibit the IGF receptor type I signal transduction function. *Molecular Cancer Therapeutics*.

[89] Rodon J, Patnaik A, Stein M (2007). A phase I man monoclonal antibody IGF-1R antagonist in patients with advanced cancer. *Journal of Clinical Oncology*.

[90] Higano CS, Yu EY, Whiting SH (2007). A phase I, first in man study of weekly IMC-A12, a fully human insulin like growth factor-1 receptor IgG1 monoclonal antibody, in patients with advanced solid tumors. *Journal of Clinical Oncology*.

[91] Tolcher AW, Rothenberg ML, Rodon J (2007). A phase I pharmacokinetic and pharmacodynamic study of AMG 479, a fully human monoclonal antibody against insulin-like growth factor type 1 receptor (IGF-1R), in advanced solid tumors. *Journal of Clinical Oncology*.

[92] Atorzi F, Tabernero J, Cervantes A (2008). A phase I, pharmacokinetic (PK) and pharmacodynamic (PD) study of weekly (qW) MK-0646, an insulin-like growth factor-1 receptor (IGF1R) monoclonal antibody (MAb) in patients (pts) with advanced solid tumors. *Journal of Clinical Oncology*.

[93] Atzori F, Tabernero J, Cervantes A (2008). A phase I, pharmacokinetic (PK) and pharmacodynamic (PD) study of weekly (qW) MK-0646, an insulin-like growth factor-1 receptor (IGF1R) monoclonal antibody (MAb) in patients (pts) with advanced solid tumors. *Journal of Clinical Oncology*.

[94] Carmeliet P (2000). Mechanisms of angiogenesis and arteriogenesis. *Nature Medicine*.

[95] Risau W, Flamme I (1995). Vasculogenesis. *Annual Review of Cell and Developmental Biology*.

[96] Lee TH, Bolontrade MF, Worth LL, Guan H, Ellis LM, Kleinerman ES (2006). Production of VEGF165 by Ewing’s sarcoma cells induces vasculogenesis and the incorporation of CD34 stem cells into the expanding tumor vasculature. *International Journal of Cancer*.

[97] Guleng B, Tateishi K, Ohta M (2005). Blockade of the stromal cell-derived factor-1/CXCR4 axis attenuates in vivo tumor growth by inhibiting angiogenesis in a vascular endothelial growth factor-independent manner. *Cancer Research*.

[98] Reddy K, Zhou Z, Jia SF (2008). Stromal cell-derived factor-1 stimulates vasculogenesis and enhances Ewing’s sarcoma tumor growth in the absence of vascular endothelial growth factor. *International Journal of Cancer*.

[99] Lindsay CR, MacPherson IRJ, Cassidy J (2009). Current status of cediranib: the rapid development of a novel anti-angiogenic therapy. *Future Oncology*.

[100] Teicher BA, Holden SA, Ara G (1995). Influence of an anti-angiogenic treatment on 9L gliosarcoma: oxygenation and response to cytotoxic therapy. *International Journal of Cancer*.

[101] Teicher BA, Holden SA, Ara G, Dupuis NP, Goff D (1995). Restoration of tumor oxygenation after cytotoxic therapy by a perflubron emulsion/carbogen breathing. *The Cancer Journal from Scientific American*.

[102] Teicher BA (1998). Role of angiogenesis in the response to anticancer therapies. *Drug Resistance Updates*.

[103] Devineni D, Klein-Szanto A, Gallo JM (1996). Uptake of temozolomide in a rat glioma model in the presence and absence of the angiogenesis inhibitor TNP-470. *Cancer Research*.

[104] Dickson PV, Hamner JB, Sims TL (2007). Bevacizumab-induced transient remodeling of the vasculature in neuroblastoma xenografts results in improved delivery and efficacy of systemically administered chemotherapy. *Clinical Cancer Research*.

